# Physeal breach as a potential predictor of pulmonary metastasis in paediatric osteosarcoma

**DOI:** 10.3389/fonc.2026.1756381

**Published:** 2026-05-05

**Authors:** Zichen Lin, Peng Huang, Jing Shan, Zhi Qi, Miaoyang Liang, Bixuan Cao, Bo Ning

**Affiliations:** 1 Department of Orthopaedics Surgery, National Children’s Medical Centre, Children’s Hospital of Fudan University, Shanghai, China; 2 School of Pharmacy, The University of Sydney, Sydney, NSW, Australia; 3 Department of Orthopaedics, The Third Affiliated Hospital of Anhui Medical University, The First People’s Hospital of Hefei, Hefei, Anhui, China

**Keywords:** imaging biomarker, metastasis, osteosarcoma, paediatric oncology, physis

## Abstract

**Background:**

Osteosarcoma (OS) is the most common primary malignant bone tumour in adolescents, and pulmonary metastasis remains the leading cause of death. In skeletally immature patients, the avascular physis is considered a barrier to local tumour spread, but its relationship to lung metastasis has not been quantified.

**Methods:**

We conducted a single-centre, retrospective cohort study of 32 paediatric OS patients. Tumour–physis relationships were evaluated before and after chemotherapy using standard local staging and chest CT. The primary outcome was pulmonary metastasis at last follow-up, analysed with multivariable logistic regression including age, sex, tumour size, location, laterality, and physeal breach. A complementary orthotopic tibial xenograft model was established by injecting 143B cells into nude mice, creating physeal-breach and non-breach groups. Vascular endothelial growth factor (VEGF) immunohistochemistry (IHC) assessed angiogenic activity at the tumour–physis interface.

**Results:**

Pulmonary metastases were present in 22% at diagnosis and in 50% (16/32) by last follow-up. Physeal breach was the only independent predictor of metastasis (odds ratio 59.89; 95% CI 3.34–1073.95; p=0.006). In the xenograft model, pulmonary metastases developed in all physeal-breach mice and in none of the non-breach group. VEGF IHC showed increased angiogenic activity in breach-associated tumours.

**Conclusion:**

Physeal breach identifies a biologically aggressive subset of paediatric OS with high metastatic potential. Because breach status is readily appreciable on routine imaging, it may serve as a practical biomarker to refine risk stratification, predict pulmonary metastasis and guide evaluation of anti-angiogenic strategies.

## Introduction

Osteosarcoma (OS) is the most common primary malignant bone tumour of adolescence, and metastatic dissemination—predominantly to the lungs—remains the principal driver of mortality despite modern multimodal therapy (1, 2). Five-year survival exceeds 60% for localized disease but remains poor once metastases are present (1). Approximately 15–20% of patients present with detectable pulmonary metastases at diagnosis, and many more harbor micro metastatic disease that later declares itself clinically (1). These observations underscore the need to identify anatomic or microenvironmental features that modulate the rate of dissemination (1, 3).

Conventional OS usually arises in the metaphysis of long bones, most often around the knee (1, 4). This characteristic topography brings the advancing tumour front into close apposition with the physis and epiphysis in skeletally immature patients, raising practical questions about local containment, joint-preserving surgery, and potential implications for metastatic spread (4).

For decades, the physis has been viewed as a relative, not absolute, barrier to local transphyseal extension of metaphyseal malignancies (4–6). Histopathologic–imaging correlation studies have documented epiphyseal involvement in a substantial subset of paediatric OS when the primary tumour abuts the physis, with magnetic resonance imaging (MRI) reliably depicting this pattern (5, 6). Conversely, in many cases the epiphysis remains uninvolved, allowing epiphyseal-sparing resections without excess local failure (4, 6). Collectively, these data establish that the physis may sometimes delay or deter local extension, but that invasion does occur under specific biological and anatomical conditions (4–6).

Several lines of evidence suggest plausible mechanisms by which an open physis might also delay systemic dissemination. First, normal hyaline cartilage is avascular, and classic anatomic studies describe separate metaphyseal and epiphyseal vascular trees with no direct intraosseous anastomoses traversing an intact physis in children (7). Second, the cartilage extracellular matrix is enriched in endogenous anti-angiogenic factors, creating a microenvironment that resists neovascularization and cellular invasion (8, 9). Third, physeal involution at puberty gradually re-establishes vascular communications across the metaphyseal-epiphyseal junction, and OS-driven angiogenesis and osteoclastic remodelling can focally erode the physis, together reducing this barrier (1, 7). Finally, recent work has identified specialized intercondylar transphyseal vascular-canal complexes in some immature knees, highlighting anatomical heterogeneity and explaining focal “breakpoints” where tumours may cross (10).

Despite this mechanistic rationale and the literature on local transphyseal invasion, whether physeal breach identified on routine restaging imaging predicts pulmonary metastasis in paediatric osteosarcoma has not been quantified. We therefore evaluated the association between post-chemotherapy physeal breach and pulmonary metastasis in a single-centre retrospective cohort using multivariable logistic regression adjusted for key clinical covariates. To provide biologic plausibility, we complemented the clinical analysis with an orthotopic 143B-Luc tibial xenograft model comparing physeal-breach versus non-breach implantation and assessed VEGF expression at the tumour–physis interface by immunohistochemistry.

## Material and methods

### Study design, setting, and eligibility

**Figure 1:**
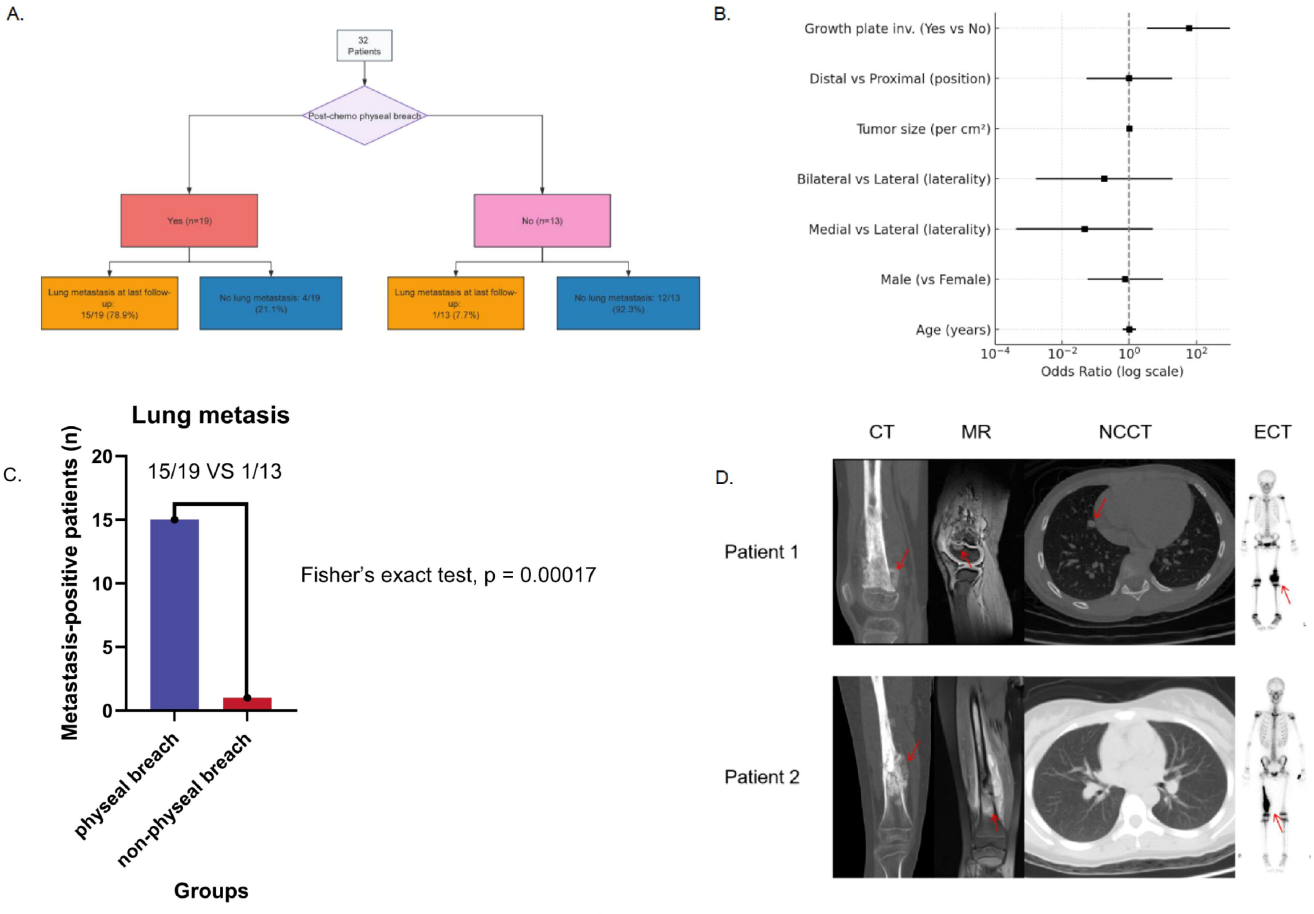
Physeal breach predicts pulmonary metastasis in paediatric osteosarcoma. **(A)** Study flow by post-chemotherapy physeal status: 19 patients had a breach (15/19 [78.9%] with lung metastasis), and 13 had no breach (1/13 [7.7%] with metastasis). **(B)** Multivariable logistic regression shows physeal breach as the only independent predictor of pulmonary metastasis (OR 59.89; 95% CI 3.34–1073.95; p = 0.006). **(C)** Pulmonary metastasis-positive patients (n) by physeal breach status (15/19 vs 1/13; yes/no). Fisher’s exact test, p = 0.00017. **(D)** Representative imaging: Breach—MRI shows transphyseal extension; CT reveals lung nodules; bone scintigraphy shows tracer crossing the physis. No breach—tumour abuts but does not cross the physis; CT shows no nodules. Arrows indicate key findings; CT, computed tomography; MRI, magnetic resonance imaging; NCCT, non-contrast chest CT; ECT, emission computed tomography.

This single-centre, retrospective observational cohort included consecutive paediatric and adolescent patients (≤18 years) with histologically confirmed long-bone osteosarcoma who received neoadjuvant chemotherapy, interval restaging, definitive surgery, and surveillance. This study duration is from Jan 1^st^, 2020 to August 1^st^, 2025 (**Figure 1A**). Data were abstracted into a standardized spreadsheet; the analytic cohort comprised 32 patients with variables summarized in **Tables 1**, **2**, **3**. Inclusion required pre-treatment, local staging and chest imaging, post-chemotherapy restaging prior to surgery, and follow-up imaging. Patients were included irrespective of lung metastasis at presentation.

**Table 1:** Baseline demographic, tumour, metastasis, and follow-up characteristics of the 32 paediatric osteosarcoma patients.

Patient ID	Age	gender	Tumor laterality	Tumor size	Tumor position	Pre-treatment Lung Metastasis	Isotope involvement of growth plate	Lung Metastasis (Post-chemotherapy)	Growth Plate Involvement (Post-chemotherapy)	Lung Metastasis at Last Follow-up	Follow-up duration (months)
01	13y6m	Female	Bilateral	5.35*4.04	Right distal femur	No	No	Yes	Yes	Yes	3
02	13y3m	Female	Bilateral	8.16*23.67	Right distal femur	No	Yes	No	Yes	Yes	34
03	16y7m	Male	Bilateral	4.55*14.02	Right distal femur	No	No	No	Yes	Yes	22
04	13y7m	Male	Lateral	8.27*7.31	Right distal femur	No	Yes	No	Yes	Yes	24
05	9y2m	Female	Medial	14.13*3.85	Right distal femur	No	No	No	Yes	Yes	15
06	13y6m	Female	Medial	4.19*2.33	Right proximal tibia	No	No	No	Yes	Yes	22
07	11y9m	Female	Bilateral	10.13*8.64	Left distal femur	Yes	Yes	Yes	Yes	Yes	14
08	13y4m	Male	Bilateral	8.1*9.07	Left distal femur	No	Yes	Yes	Yes	Yes	8
09	8y10m	Male	Bilateral	9.17*24.03	Left humerus (entire bone)	No	Yes	Yes	Yes	Yes	18
10	15y9m	Male	Lateral	10.53*6.41	Left distal femur	Yes	Yes	Yes	Yes	Yes	15
11	11y1m	Male	Lateral	9.97*4.64	Left distal femur	Yes	Yes	Yes	Yes	Yes	10
12	12y8m	Female	Bilateral	9.02*12.99	Right distal femur	Yes	Yes	Yes	Yes	Yes	15
13	14y11m	Female	Bilateral	22.85*7.64	Right distal femur	Yes	No	Yes	No	Yes	13
14	11y6m	Male	Bilateral	24.71*10.68	Right distal femur	Yes	Yes	Yes	Yes	Yes	4
15	13y9m	Male	Bilateral	6.08*15.74	Left proximal humerus	No	Yes	No	Yes	Yes	15
16	9y4m	Female	Bilateral	7.25*14.12	Left proximal humerus	Yes	Yes	Yes	Yes	Yes	4
17	16y10m	Female	Medial	3.46*14.44	Right proximal femur	No	No	No	No	No	12
18	17y0m	Female	Medial	3.15*5.47	Right distal femur	No	No	No	No	No	14
19	15y8m	Female	Bilateral	9.19*12.45	Left proximal humerus	No	Yes	No	Yes	No	10
20	17y0m	Male	Lateral	7.71*2.83	Left distal tibia	No	Yes	No	No	No	29
21	12y5m	Female	Medial	5.23*10.04	Left distal femur	No	Yes	No	No	No	41
22	15y9m	Male	Medial	9.45*7.21	Left distal femur	No	Yes	No	Yes	No	23
23	11y9m	Male	Bilateral	24.28*2.66	Left mid femur	No	No	No	No	No	11
24	13y4m	Female	Medial	17.28*8.18	Right distal femur	No	Yes	No	No	No	14
25	12y1m	Female	Bilateral	19.71*9.95	Left distal femur	No	Yes	No	No	No	13
26	7y11m	Male	Bilateral	14.34*7.35	Right distal femur	No	No	No	No	No	9
27	14y6m	Female	Medial	13.52*8.23	Left distal femur	No	Yes	No	No	No	58
28	8y2m	Female	Bilateral	9.1*6.71	Right distal femur	No	No	No	Yes	No	17
29	10y11m	Male	Bilateral	13.57*4.43	Right distal femur	No	No	No	No	No	19
30	11y9m	Female	Medial	6.95*9.05	Left distal femur	No	No	No	No	No	10
31	13y9m	Female	Medial	6.14*7.63	Left distal femur	No	No	No	Yes	No	12
32	10y9m	Female	Medial	4.52*7.08	Left proximal tibia	No	No	No	No	No	4

Each row represents one patient and reports age, sex, tumour laterality and location, tumour size, pulmonary metastasis status (pre-treatment, post-chemotherapy, and at last follow-up), radionuclide evidence of growth plate involvement, physeal breach (post-chemotherapy), and follow-up duration (months; from diagnosis to the last documented clinical visit and/or surveillance chest imaging). Abbreviations: y, year; m, month.

In the Tumor size column, the symbol ‘*’ indicates multiplication between the longest tumor diameter and the perpendicular diameter.

**Table 2:** Comparison of clinical and tumour characteristics between patients with and without pulmonary metastasis at last follow-up.

Variables	Pulmonary metastasis	Non-pulmonary metastasis	P-value
Patients, n	16	16	
Age(mean+SD, y)	12y8m ± 2y3m	13y1m ± 2y11m	0.635
Gender, n			0.473
Male	8	5	
Female	8	11	
Tumor laterality, n			0.031
Lateral	3	1	
Medial	2	9	
Bilateral	11	6	
Tumor size(mean+SD, cm^2)	103.25 ± 72.93 cm²	75.36 ± 47.27 cm²	0.21
Tumor position			1.0
Distal	13	12	
Proximal	3	4	
Physeal breach			0.00017
Yes	15	4	
No	1	12	
Follow-up duration (months), median (IQR) [range]	15.0 (IQR 9.5–19.0) [3–34]	13.5 (IQR 10.75–20.0) [4–58]	0.88

Patients were stratified into pulmonary-metastasis and non-pulmonary-metastasis groups (n = 16 each). Categorical variables are presented as n and compared using Fisher’s exact test or χ² test, as appropriate. Continuous variables are presented as mean ± SD or median (IQR) [range] and compared using Student’s t test or the Mann–Whitney U test, as appropriate. Follow-up duration (months) was calculated from diagnosis to the last documented clinical visit and/or surveillance chest imaging. Abbreviations: IQR, interquartile range; y, year; m, month.

**Table 3:** Multivariable logistic regression analysis identifying independent predictors of pulmonary metastasis by last follow-up.

Variable	Coefficient (log-odds)	SE	OR	95% CI	p-value
Age (years)	0.01	0.24	1.01	0.64–1.61	0.97
Male (vs Female)	–0.27	1.32	0.76	0.06–10.16	0.84
Tumour laterality: Medial (vs Lateral)	–3.06	2.39	0.05	0.0004–5.07	0.2
Tumour laterality: Bilateral (vs Lateral)	–1.73	2.39	0.18	0.002–18.93	0.47
Tumour size (per cm²)	0.01	0.01	1.01	0.99–1.04	0.34
Tumour position: Distal (vs Proximal)	0	1.49	1	0.05–18.53	1
Physeal breach (Yes vs No)	4.09	1.47	59.89	3.34–1073.95	0.006

Physeal breach was the only independent predictor of pulmonary metastasis (odds ratio 59.89, 95% CI 3.34–1073.95, p = 0.006). Other factors (age, sex, tumour laterality, tumour size, and tumour location) did not show significant independent associations with metastasis (each p > 0.2).

### Patient consent and ethics statement

The study was approved by the Ethics Committee of the Children’s Hospital of Fudan University (Approval No. 2022-202) and was conducted in accordance with the Declaration of Helsinki and all relevant institutional and national regulations. Written informed consent was obtained from all participants or their legal guardians.

### Imaging acquisition and interpretation

Local tumour extent was assessed using standard clinical imaging at baseline and after neoadjuvant chemotherapy per institutional practice. A baseline isotope bone scan documented whether tracer uptake crossed the physis. Chest computed tomography (CT) was performed at baseline, after neoadjuvant chemotherapy, and during surveillance. Abstracted imaging variables included tumour laterality (lateral/medial/bilateral), tumour position (distal vs proximal), and tumour size recorded as longest dimension × perpendicular dimension and summarized analytically as area (**Table 1** and **Table 2**). Physeal breach was reassessed on post-chemotherapy restaging.

### Treatment, follow-up, and outcomes

Patients were managed along standard-of-care pathways comprising multi-agent neoadjuvant chemotherapy and/or surgery, followed by surveillance with periodic chest CT and local imaging. The primary outcome was presence of pulmonary metastases at last follow-up, which also defined the “pulmonary metastases” versus “non-pulmonary-metastases” groups used in comparative analyses. Secondary timepoints included lung metastasis at presentation (pretreatment) and after neoadjuvant chemotherapy (post-chemotherapy). Physeal breach was assessed at baseline isotope study and post-chemotherapy restaging.

### Cell-derived xenograft tumour model experiments

Female BALB/c nude mice (4 weeks old; ~20 g) were obtained from the Shanghai Jiao Tong University Animal Research Centre (Shanghai, China) and maintained under specific pathogen-free (SPF) conditions. Under general anaesthesia with isoflurane in oxygen (4–5% induction, 2–3% maintenance), each mouse received an orthotopic inoculation of 1 × 10^6 luciferase-transfected 143B cells (143B-Luc) suspended in 50 μL PBS into the proximal tibia. The 143B cell line was selected as a well-characterised, metastasis-competent osteosarcoma model for orthotopic tibial implantation, allowing us to isolate the effect of physeal barrier disruption on metastatic dissemination. Mice were assigned to two groups based on the implantation method: the physeal-breach group (tumour inoculated across the physis, n = 4) and the non-physeal-breach group (tumour inoculated without crossing the physis, n = 4).

### Inoculation technique and verification of physeal breach

**Figure 2:**
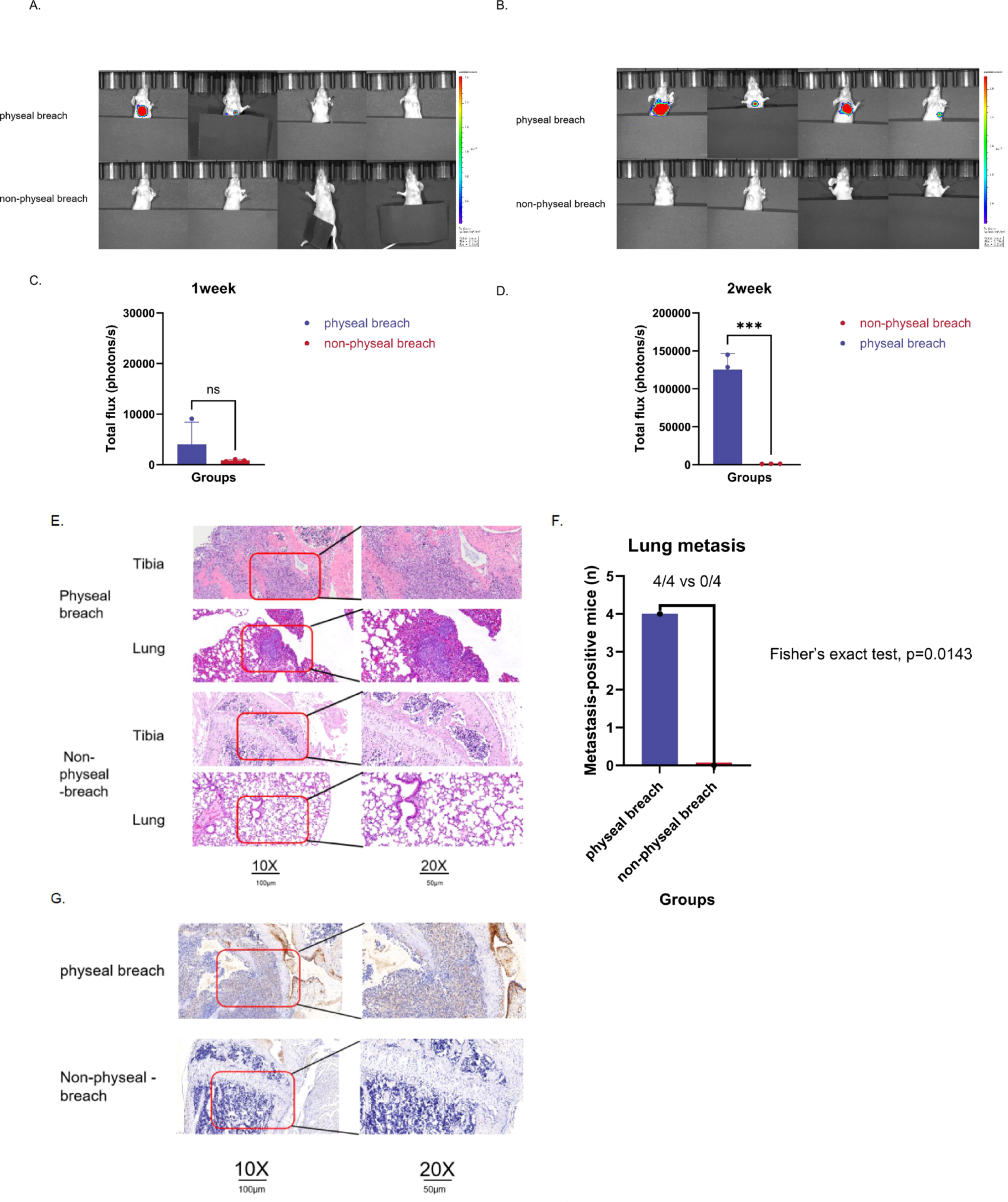
Physeal breach co-segregates with pulmonary metastasis and VEGF upregulation in an orthotopic osteosarcoma model. Female nude mice received orthotopic tibial inoculation of 1 × 10^6^ 143B cells and were assigned to physeal-breach or non-breach groups (n = 4 each). **(A, B)** Representative *in vivo* BLI images at 1 and 2 weeks; ROIs are shown as coloured overlays with pseudocolor scales at right. **(C, D)** Primary-site BLI signal (total flux, photons/s): no difference at week 1 (p = 0.2755), but higher signal in the breach group at week 2 (p =0.0005). Data are mean ± SD (n = 4 per group); comparisons by unpaired two-tailed t test (or Mann–Whitney U test if non-normal). **(E)** H&E staining of tibia and lung: the breach group shows transphyseal invasion and lung metastases, whereas the non-breach group retains an intact physis and clear lungs. Red boxes mark magnified areas (scale bars, 100 µm [10×] and 50 µm [20×]). **(F)** Incidence of pulmonary metastasis: 4/4 mice in the breach group vs 0/4 in the non-breach group (Fisher’s p = 0.0143). **(G)** VEGF IHC at the tumour–physis interface shows strong cytoplasmic/perivascular staining in breach tumours and weak/absent staining in non-breach tumours (scale bars as in E). ***p < 0.001.

To ensure accurate group assignment despite the small size of the mouse tibia, distinct injection trajectories and anatomical landmarks were used. For the physeal-breach group, the needle was introduced from the tibial plateau and advanced along the long axis of the tibia to intentionally traverse the physis. For the non-physeal-breach group, the needle was inserted distal to the physis and advanced along the short axis into the metaphyseal region to avoid traversing or damaging the growth plate. Successful group assignment was verified histologically: harvested tibias were sectioned through the physis, and H&E staining was used to confirm whether the tumour track/tissue breached the growth plate (representative images shown in **Figure 2E**).

### *In vivo* bioluminescence imaging and definition of pulmonary metastasis

The development of pulmonary metastasis was monitored longitudinally *in vivo* by bioluminescence imaging (BLI) using an IVIS imaging system (IVIS Spectrum, PerkinElmer). Mice were anaesthetised with isoflurane in oxygen and administered D-luciferin (150 mg/kg, i.p.) prior to imaging. Imaging was performed 10 min after D-luciferin administration at weekly intervals (weeks 1 and 2 post inoculation) under identical acquisition settings. Pulmonary metastasis was defined as the appearance of a discrete thoracic bioluminescent signal on serial imaging, distinct from the primary tibial tumour signal.

### Study endpoint, humane endpoints, and tissue collection

Animals were euthanised if predefined humane endpoints were met (marked reduction in activity, >20% body-weight loss, severe cachexia, or any tumour dimension exceeding 25 mm). The experiment was terminated when all mice in one group reached the prespecified study endpoint of imaging-defined pulmonary metastasis (or earlier if predefined humane endpoints were met). In this experiment, the study endpoint was reached at day 14 (week 2) post inoculation, prompting terminal collection. Euthanasia was performed by CO_2_ inhalation (100% CO_2_ introduced at 30–70% of the chamber volume per minute into a chamber initially filled with room air, i.e., not pre-filled with CO_2_) until respiration ceased, followed by cervical dislocation as a secondary physical method to ensure death. Lungs, affected tibias, and primary tumours were harvested, fixed in formaldehyde, and processed into paraffin blocks. Lung metastases detected by BLI were confirmed at necropsy by histology (H&E). All animal procedures were approved by the Animal Ethics Committee of Shanghai Jiao Tong University (Approval No. A2024372) and performed by trained personnel to minimise pain and distress.

### BLI intensity analysis

For quantitative analysis of the bioluminescent signal, regions of interest (ROIs) were drawn over the primary tibial lesion and a background area using the IVIS manufacturer’s software. Signal intensity was recorded as total flux (photons/s) (or radiance, as output by the system) after background subtraction and compared between groups at week 1 and week 2. Data are presented as mean ± SD (n = 4 per group).

### Hematoxylin and eosin staining ex vivo

Paraffin-embedded lung and tibial sections were deparaffinized in xylene for 20 min, then rehydrated through graded ethanol (100%, 90%, 75%) and rinsed in tap water. Frozen sections were brought to room temperature for 5–10 min, fixed as required, and rinsed. Slides were stained with hematoxylin for 3–5 min, rinsed, differentiated in hematoxylin differentiation solution (Cat. G1039, Servicebio, China) for 2–5 s, and rinsed again. Bluing was performed with hematoxylin bluing solution (Cat. G1040, Servicebio, China) for 2–5 s, followed by eosin staining for 5 min after ethanol dehydration. Sections were then cleared in xylene, mounted with neutral balsam, and cover slipped. Slides were evaluated using a whole-slide tissue scanner (Pannoramic MIDI II, 3DHISTECH^®^, Hungary).

### Immunohistochemistry

Formalin-fixed, paraffin-embedded tumour sections (4 µm) underwent deparaffinization, rehydration, heat-induced epitope retrieval (citrate buffer, pH 6.0), and peroxidase blockade (3% H_2_O_2_). Sections were blocked and incubated with primary antibody to the marker of interest at manufacturer-recommended dilutions, followed by HRP-conjugated secondary antibody and DAB chromogen. Slides were counterstained with hematoxylin, dehydrated, and mounted. Negative controls omitted the primary antibody; appropriate positive controls were included.

### Statistical analysis

Statistical analyses were performed using GraphPad Prism (version 10.6.1; GraphPad Software, San Diego, CA, USA) and R (R Foundation for Statistical Computing, Vienna, Austria) in RStudio (version 2026.01.0 + 392; Posit Software, Boston, MA, USA). GraphPad Prism was used for descriptive statistics and group comparisons, and multivariable logistic regression was performed in R. Age recorded as years and months was converted to years for analysis. Continuous variables were inspected for distributional assumptions; summary statistics are presented as mean ± SD (e.g., tumour area in cm²). Group comparisons between patients with versus without pulmonary metastases at last follow-up used two-sided tests with α = 0.05: Student’s t-test (or Mann-Whitney U for non-normal distributions) for continuous variables and χ² test or Fisher’s exact test for categorical variables. Independent associations with the primary outcome were estimated using multivariable logistic regression as appropriated. Results are reported as coefficients (log-odds), standard errors, odds ratios (ORs), 95% confidence intervals (CIs), and p-values.

## Results

### Patient demographics and tumour characteristics

A total of 32 patients were included, with a median age of 13 years (range, 8–17 years). The majority were female (19 patients, 59%). Tumours were predominantly located in distal bone segments (25 of 32, 78%), mainly the distal femur and tibia, with only 7 tumours (22%) arising in proximal locations. Tumour size varied widely (two-dimensional area, 10–264 cm²; mean ± SD, 89 ± 60 cm²). At diagnosis, 7 patients (22%) presented with pulmonary metastases. With respect to laterality, 17 patients (53%) showed bilateral involvement of the medial and lateral epiphyseal aspects, 11 (34%) had medial-only tumours, and 4 (13%) had lateral-only tumours (**Table 1**).

### Comparison of patients with versus without pulmonary metastasis

Among the 32 patients, 16 (50%) developed pulmonary metastasis (either present at diagnosis or during follow-up), whereas 16 (50%) remained metastasis-free. The mean age was comparable between groups (12.7 vs 13.1 years; p = 0.635), and sex distribution did not differ significantly (female, 50% vs 69%; p = 0.473). Tumour laterality differed significantly by metastasis status (p = 0.031). Patients with pulmonary metastasis were more likely to have tumours involving both medial and lateral epiphyseal aspects (11/16, 68.8%) compared with non-metastatic cases (6/16, 37.5%). Conversely, isolated medial condylar tumours were less common in the metastasis group (2/16, 12.5%) than in the non-metastatic group (9/16, 56.3%), while lateral-only tumours were infrequent in both groups (3 vs 1 case). Tumour size was larger on average in the metastasis group (mean 103.3 ± 72.9 cm²) than in the non-metastasis group (75.4 ± 47.3 cm²), but the difference was not statistically significant (p = 0.210). Tumour location (distal vs proximal) was comparable between groups (distal 81% vs 75%; p = 1.00). Physeal breach was strongly associated with pulmonary metastasis: 15 of 16 patients (93.8%) with metastasis exhibited physeal breach, compared with only 4 of 16 (25%) among those without metastasis (p = 0.00017). This difference is illustrated in **Figure 1C**. In summary, physeal breach was nearly universal among patients who developed pulmonary metastasis but absent in most metastasis-free cases (**Table 2**). Follow-up duration did not differ between patients with versus without pulmonary metastasis at last follow-up (**Table 2**, p = 0.88) and was also similar between patients with versus without physeal breach (**Supplementary Table 3**, p = 0.985).

### Logistic regression analysis

In multivariable logistic regression (**Table 3**, **Figure 1B**), physeal breach emerged as the only significant independent predictor of pulmonary metastasis. Patients with physeal breach had markedly higher odds of developing metastases (OR 59.89, 95% CI 3.34–1073.95, p = 0.006). None of the other variables retained statistical significance in the adjusted model. Age, sex, tumour laterality, tumour size, and tumour location all showed no independent association with metastasis. Collectively, these findings demonstrate that physeal breach is strongly and independently associated with pulmonary metastasis, whereas other demographic and tumour-related characteristics exerted no significant influence on metastatic risk.

### Representative imaging of patients and H&E histology from mouse model: physeal breach with pulmonary metastasis versus non-physeal breach without metastasis

Representative clinical imaging of two patients illustrates the prognostic contrast at the tumour–physis interface. Patient 1 (**Figure 1D**) exhibited clear transphyseal extension with epiphyseal involvement (physeal breach) and developed pulmonary metastasis. Patient 2 (**Figure 1D**) had a tumour abutting the physis without breach (non-physeal breach) and no pulmonary metastasis. Hematoxylin and eosin (H&E) histology corroborated these findings in the mouse model. In mice with physeal breach (**Figure 2E** and **Supplementary Figure S1A**), the upper panel shows malignant permeation across the physeal cartilage, and the lower panel demonstrates metastatic nodules within the lung parenchyma. In contrast, in mice with non-physeal breach, the upper panel displays a clear tumour–physis boundary, and the lower panel shows normal lung tissue without metastatic lesions.

### *In vivo* BLI and metastatic outcome by physeal status

Representative live-animal optical images demonstrated focal, lesion-localized BLI at the primary site under standardized acquisition parameters, with corresponding quantification of mean BLI intensity (**Figures 2A–D**). Mice with physeal breach exhibited evident pulmonary metastases, whereas those with non-physeal breach showed no detectable metastases during the first week. By the second week, physeal status co-segregated with metastatic outcome: all mice with documented physeal breach developed pulmonary metastases (4/4), whereas none with non-physeal breach did so (0/4), indicating a significant association between physeal breach and metastatic dissemination (p = 0.0143; **Figure 2F** and **Supplementary Table 1**).

### VEGF immunohistochemistry at the tumour–physis interface

To assess angiogenic activity at the tumour–physis boundary, VEGF immunohistochemistry (IHC) was performed on harvested tibial tumour sections from the orthotopic model. VEGF staining at the tumour–physis interface appeared qualitatively stronger in the physeal-breach group than in the non-breach group (**Figure 2G** and **Supplementary Figure S1B**), showing prominent cytoplasmic/perivascular immunoreactivity in breach-associated tumours. This VEGF-enriched interface was observed alongside the higher incidence of pulmonary metastasis in breach-group mice (4/4 vs 0/4 by week 2). VEGF IHC was assessed descriptively in this study and no formal quantitative scoring was performed.

## Discussion

In this single-centre paediatric cohort of 32 patients, physeal breach emerged as the only independent predictor of pulmonary metastasis. Univariable analysis showed that 93.8% of patients who developed lung metastases had physeal breach compared with only 25.0% of those who did not. Representative imaging and histology illustrated these extremes, while *in vivo* optical imaging and xenograft data confirmed that physeal breach co-segregated with metastasis. Together, these observations suggest that breach functions as a pragmatic marker of systemic risk, extending beyond its traditional role in local surgical planning.

From a diagnostic perspective, accurate identification of physeal breach is crucial for staging and surgical planning. MRI remains the gold standard for evaluating the tumour–physis relationship because of its excellent soft-tissue contrast and capacity to delineate subtle transphyseal invasion (2). However, isotope bone scintigraphy (ECT) offers complementary functional information: focal tracer uptake traversing the physis may reflect early metabolic activation or angiogenic remodelling before overt structural disruption (3, 4). When interpreted alongside MRI, ECT can help detect early functional compromise of the physis and may thus reveal a biologically aggressive tumour phenotype earlier in its course.

The biological rationale for this association centres on the unique anatomy and microenvironment of the physis. In skeletally immature patients, the physis is composed of avascular hyaline cartilage that separates epiphyseal and metaphyseal vascular trees, functioning as a relative barrier to local tumour extension (4, 11). This barrier is reinforced by the cartilage extracellular matrix, which is enriched in endogenous anti-angiogenic factors that resist endothelial ingrowth and cellular invasion (8, 9). However, the barrier is not absolute. Anatomical studies have identified intercondylar transphyseal canal complexes that provide focal “breakpoints” across the physis, explaining why some tumours traverse the plate while others remain contained (10). Tumour hypoxia after rapid growth and cytotoxic stress can activate HIF-1, which in turn up-regulates vascular endothelial growth factor (VEGF) and initiates a local “angiogenic switch” at the tumour–physis frontier. VEGF drives endothelial proliferation and sprouting, increases vascular permeability (loosening the endothelial barrier), and remodels the extracellular matrix by coupling to MMP activation—particularly MMP-9—thereby liberating matrix-bound VEGF and reinforcing a feed-forward loop (12). In parallel, VEGF has chemotactic and pro-resorptive effects on osteoclasts, promoting focal cartilage/bone erosion. Within the normally avascular hyaline cartilage of the physis, this VEGF-high microenvironment erodes the barrier, permits neo vessels to penetrate physeal cartilage, and creates permissive “tracks” along which invasive osteosarcoma cells can traverse into the epiphysis and intravasate (13). Collectively, these processes provide a mechanistic bridge from physeal breach to efficient pulmonary dissemination. Consistent with this model, our data show that physeal breach is the only independent predictor of lung metastasis, and *in vivo* experiments demonstrate perfect co-segregation of breach with metastasis. In our study, VEGF immunoreactivity at the tumour–physis interface appeared stronger in mice with breach than in those with an intact physis, supporting the concept that breach is a visible correlate of a VEGF-active state (**Figure 2G** and **Supplementary Figure S1B**). These VEGF IHC findings are descriptive and hypothesis-generating, and do not establish a causal mechanism. Furthermore, a meta-analysis of 22 studies has shown that high VEGF expression in osteosarcoma is associated with worse overall survival and higher odds of metastasis, aligning molecular phenotype with systemic behaviour (14). In this context, physeal breach is not simply an anatomical descriptor but rather a visible correlate of a pro-angiogenic, VEGF-high microenvironment that enables tumour cell intravasation and efficient pulmonary dissemination. This co-segregation of breach with metastasis in our xenograft model strengthens the plausibility of this mechanistic link.

Our results extend prior literature, which has primarily emphasised the local implications of physeal invasion for epiphysis-sparing resections, by demonstrating that breach also signals systemic metastatic risk (4, 11, 15). Clinically, these findings suggest that patients with persistent breach after neoadjuvant chemotherapy may require closer thoracic surveillance, while intact physeal could justify function-preserving strategies without systemic compromise. Imaging studies reinforce the importance of MRI for assessing physeal involvement and CT as the gold standard for pulmonary metastases (2, 15). Recent radiomics and artificial intelligence approaches show promise for improving risk stratification, but reproducibility and validation remain limiting (16, 17). Therapeutically, targeting VEGF has yielded mixed results in osteosarcoma. Regorafenib improved progression-free survival in placebo-controlled trials, and cabozantinib achieved disease control in a single-arm phase II, while lenvatinib combined with ifosfamide/etoposide failed to prolong PFS in a randomised study (18–20). These outcomes underscore the need for biomarker-guided application of anti-angiogenic strategies. Breach status, reflecting a VEGF-active state, may serve as a pragmatic stratification tool for clinical trial design.

### Limitation

This study has several limitations. The clinical cohort was retrospective and single-centre, and the sample size was limited, which may reduce generalisability and statistical power. Although follow-up duration was recorded in months and added to the tables, residual follow-up bias or informative censoring (e.g., loss to follow-up or death) cannot be fully excluded in a chart-based study. Pulmonary metastasis was assessed on routine chest imaging and analysed as a binary outcome (presence/absence), rather than metastatic burden, which may introduce misclassification and limits time-to-event interpretation. The multivariable logistic model is limited by the small number of metastatic events relative to the number of parameters, resulting in wide confidence intervals and potential small-sample bias; thus, the estimated magnitude of association should be interpreted cautiously and requires external validation.

The orthotopic xenograft experiment was designed as a pilot study with a small number of animals (n = 4 per group). In line with the 3R principle (Reduction) and practical resource constraints, this small n limits statistical power and the precision of effect estimation. Therefore, the animal findings are presented as supportive evidence rather than definitive proof. The prespecified terminal criterion was imaging-defined pulmonary metastasis (or predefined humane endpoints if reached earlier), and in this experiment terminal collection occurred at week 2 (day 14); thus, later-onset metastasis in the non-breach group cannot be excluded with longer follow-up, and findings may vary across osteosarcoma models and cell lines.

## Conclusion

Across this single-centre paediatric cohort and the orthotopic model, physeal breach emerged as the sole independent predictor of pulmonary metastasis, with a VEGF-rich tumour–physis interface providing biologic plausibility. Because breach status is readily appreciable on routine restaging imaging, it can be integrated into care to refine risk stratification, serve as a predictor of pulmonary metastasis, and guide decisions on epiphysis-sparing surgery. Prospective multicentre validation and biomarker-guided trials—particularly targeting angiogenic pathways—are warranted to test whether breach-positive patients benefit from intensified systemic strategies.

## Data Availability

The original contributions presented in the study are included in the article/**Supplementary Material**. Further inquiries can be directed to the corresponding author.

## Supplementary material

Supplementary Figure 1 Ex vivo H&E staining and VEGF immunohistochemistry.

Supplementary Table 1 Imaging-defined pulmonary metastasis outcomes in the orthotopic osteosarcoma mouse model (week 1 and week 2), stratified by physeal breach status.

Supplementary Table 2 Sensitivity analyses addressing potential follow-up length bias (follow-up ≥6 months and ≥12 months).

Supplementary Table 3 Follow-up duration stratified by physeal breach status.
