# Supplementary material for: Physeal breach as a potential predictor of pulmonary metastasis in paediatric osteosarcoma
Source: Front Oncol. 2026 May 5;16:1756381. doi: 10.3389/fonc.2026.1756381 (PMC13183857; doi:10.3389/fonc.2026.1756381)
Supplement: Supplementary Figure 1 — Ex vivo H&E staining and VEGF immunohistochemistry. [file DataSheet1.docx]

**Supplementary Fig. 1S**

H.E and VEGF staining results. A, H.E sections of the remaining cases (physeal-breach n=3; non-breach n=3) highlighting transphyseal invasion in breach and an intact physis in non-breach (scale bars as indicated); B, VEGF immunohistochemistry of the same cases shows strong cytoplasmic/perivascular DAB staining at the tumor–physis interface in breach and weak/absent staining in non-breach.


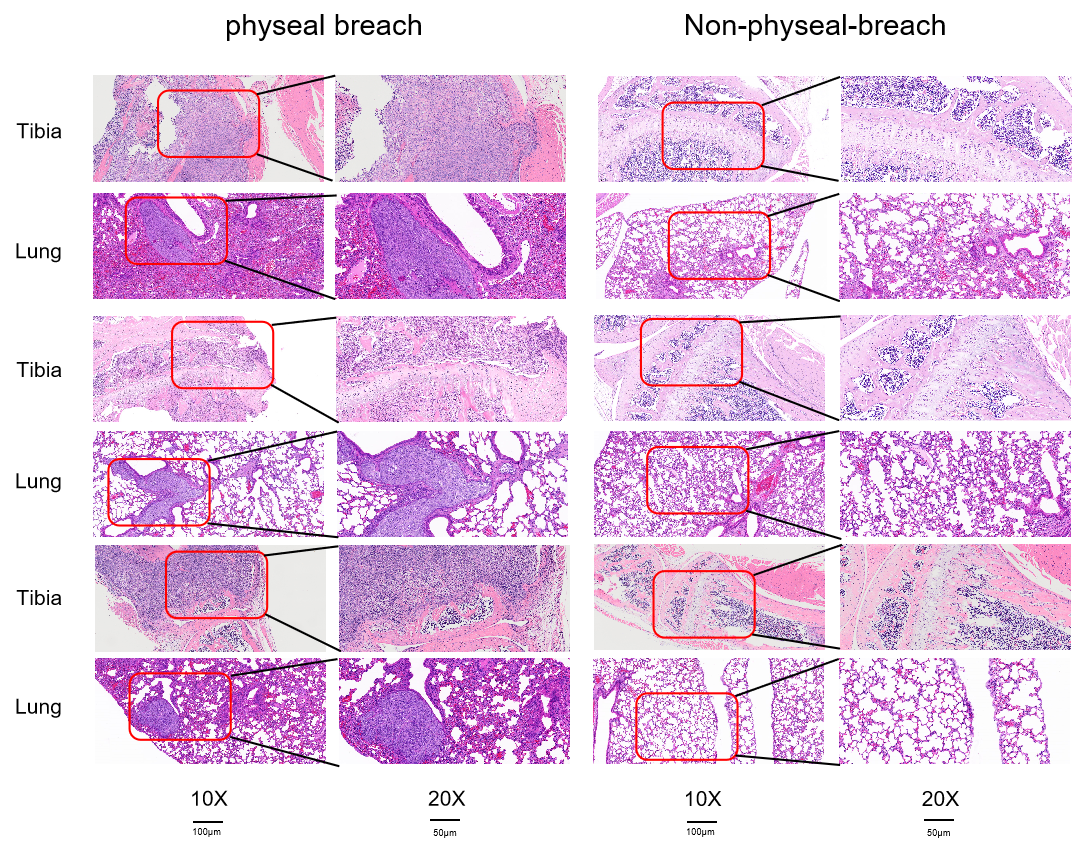
A.


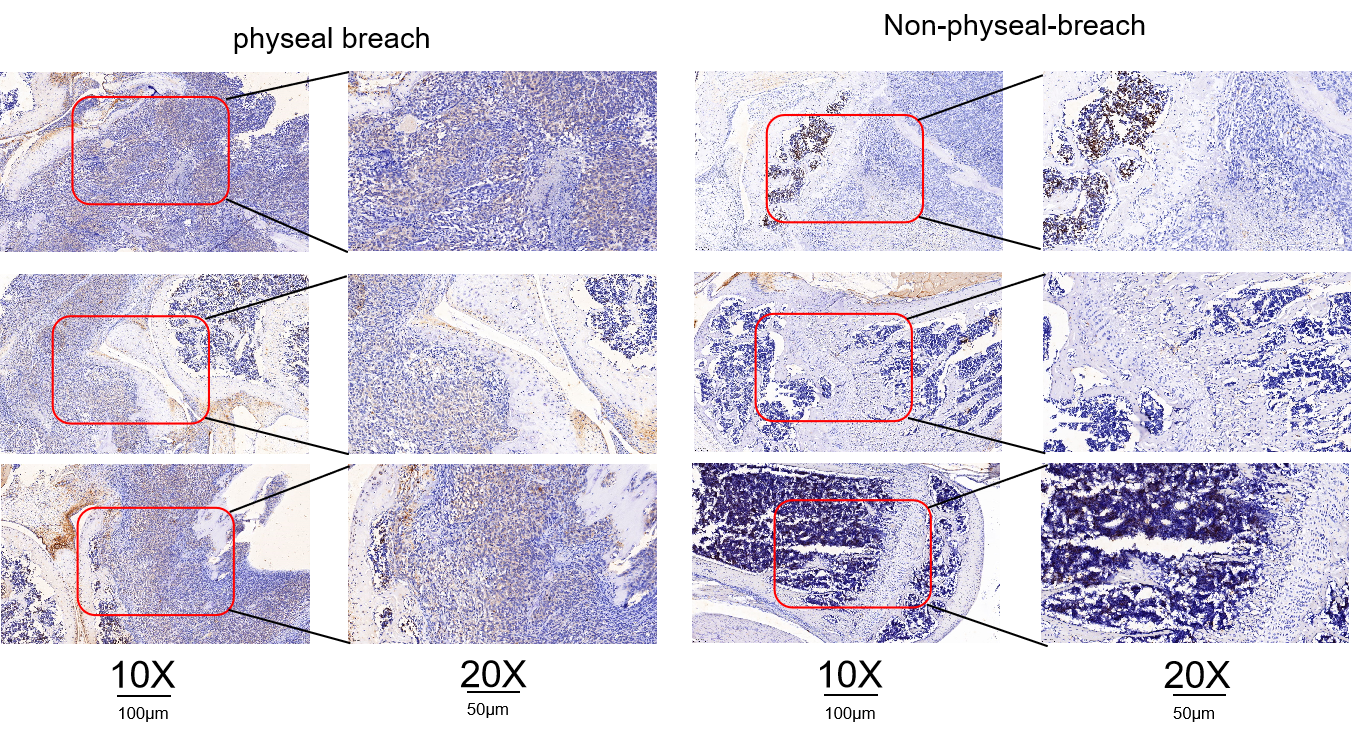


B.

**Supplementary Table. S1**

Pulmonary metastasis outcomes in an orthotopic osteosarcoma mouse model, stratified by the presence of physeal breach. At 1 week post tumor implantation, there was no significant difference: 2 of 4 mice with physeal breach had lung metastases vs 0 of 4 with intact physes (p = 0.2143). By 2 weeks, metastases developed in all 4 of 4 mice with physeal/epiphyseal breach, whereas none of the 4 mice with intact physes had metastasis, a significant association (p = 0.0143).

|  | **Lung-Metastasis** | **non-Lung-Metastasis** | **p-value** |
| --- | --- | --- | --- |
| 1 weeks |  |  |  |
| **physeal breach** | 2 | 2 | 0.2143 |
| **non-physeal breach** | 0 | 4 |  |
| 2 weeks |  |  |  |
| **physeal breach** | 4 | 0 | 0.0143 |
| **non-physeal breach** | 0 | 4 |  |

**Supplementary Table S2. Sensitivity analyses addressing potential follow-up length bias.**
The association between physeal breach (post‑chemotherapy growth plate involvement) and pulmonary metastasis at last follow-up was re-evaluated after (A) excluding patients with follow-up <6 months (follow-up ≥6 months) and (B) restricting the cohort to patients with follow-up ≥12 months. Data are presented as counts. P-values were calculated using Fisher’s exact test. Odds ratios were computed as the cross-product ratio.

A.

| **Physeal breach (post-chemo)** | **pulmonary-Metastasis** | **non-pulmonary-Metastasis** | Total |
| --- | --- | --- | --- |
| Yes | 12 | 4 | 16 |
| No | 1 | 11 | 12 |
| Total | 13 | 15 | 28 |

Fisher’s exact test: **p = 0.00064**

Odds ratio (cross‑product): **OR = 33.0**

**B.**

| **Physeal breach (post-chemo)** | **pulmonary-Metastasis** | **non-pulmonary-Metastasis** | Total |
| --- | --- | --- | --- |
| Yes | 10 | 3 | 13 |
| No | 1 | 8 | 9 |
| Total | 11 | 11 | 22 |

Fisher’s exact test: **p = 0.00752**

Odds ratio (cross‑product): **OR = 26.67**

****Supplementary Table S3. Follow-up duration stratified by physeal breach status.**Follow-up duration (months) was calculated from diagnosis to the last documented clinical visit and/or surveillance chest imaging. Data are presented as median (IQR) [range] and compared using the Mann–Whitney U test. Abbreviation: IQR, interquartile range.**

| **Variables** | **Physeal breach (Yes)** | **Physeal breach (No)** | **P-value** |
| --- | --- | --- | --- |
| Patients, n | 19 | 13 | — |
| Follow-up duration (months), median (IQR) [range] | 15.0 (10.0–20.0) [3–34] | 13.0 (11.0–19.0) [4–58] | 0.985 |

**Test: Mann–Whitney U（Wilcoxon rank-sum）**
